# Severity-adapted graded exercise rehabilitation reduces systemic inflammation and improves functional capacity in hospitalized AECOPD: an assessor-blinded randomized controlled trial

**DOI:** 10.3389/fphys.2026.1767608

**Published:** 2026-04-16

**Authors:** Hui Zeng, Yue Chen, Hongmin Ran, Dehua Zhao, Yan Wang, Dandan Fu, Nana Yang, Jimei Luo, Lina Ma, Qiuhang Hu, Lulu Huang, Changxiu Li, Luwen Luo, Rong Liu

**Affiliations:** 1 The Second Affiliated Hospital of Zunyi Medical University, Zunyi, Guizhou, China; 2 Nursing School of Zunyi Medical University, Zunyi, Guizhou, China; 3 The Affiliated Hospital of Zunyi Medical University, Zunyi, Guizhou, China

**Keywords:** AECOPD, functional capacity, graded exercise rehabilitation, inflammatory biomarkers, physiological tolerance, randomized controlled trial

## Abstract

**Background:**

Hospitalized acute exacerbation of chronic obstructive pulmonary disease (AECOPD) is characterized by heightened systemic inflammation and rapid decline in functional capacity. Although exercise rehabilitation is recommended, the physiological appropriateness of exercise intensity during acute hospitalization remains uncertain, particularly across different levels of disease severity.

**Methods:**

This prospective, assessor-blinded, randomized controlled trial enrolled 141 hospitalized patients with AECOPD. Participants were stratified by disease severity (Grade I–III) and randomized to either a severity-adapted graded exercise rehabilitation program (Study group, n=70) or conventional exercise rehabilitation (Control group, n=71) for 2 weeks from admission, in addition to standard medical treatment. The severity-adapted program applied an inverse matching strategy to optimize relative physiological load, whereby patients with more severe disease received lower-intensity exercise and those with milder disease received higher-intensity exercise. Primary outcomes were changes in systemic inflammatory biomarkers (interleukin-6 [IL-6], interleukin-8 [IL-8], tumor necrosis factor-α [TNF-α], high-sensitivity C-reactive protein [hs-CRP], and white blood cell count [WBC]). Secondary outcomes included functional capacity and symptom-related measures, assessed using the 6-minute walk test (6MWT), modified Medical Research Council dyspnea scale (mMRC), COPD Assessment Test (CAT), and Hospital Anxiety and Depression Scale (HADS).

**Results:**

Compared with conventional rehabilitation, severity-adapted graded exercise resulted in a more favorable inflammatory marker profile and greater improvements in functional capacity and symptom burden. Among the inflammatory outcomes, the most statistically robust between-group differences were observed for IL-8, TNF-α, and WBC, whereas IL-6 and hs-CRP showed directionally consistent but more modest evidence. Within the severity-adapted group, patients with milder disease tended to show larger anti-inflammatory and functional gains under higher, well-tolerated relative exercise intensity, whereas patients with moderate-to-severe disease achieved stable improvements under low-to-moderate intensity training. No consistent severity-dependent response pattern was observed in the control group.

**Conclusion:**

Severity-adapted graded exercise rehabilitation initiated during hospitalization for AECOPD was associated with a more favorable inflammatory profile and greater improvements in functional capacity and symptom-related outcomes than conventional exercise rehabilitation. These findings support a severity- and function-informed strategy for individualized exercise prescription in hospitalized patients with AECOPD.

**Clinical trial registration:**

https://www.chictr.org.cn, ChiCTR; ChiCTR2300072409.

## 1 Introduction

Chronic obstructive pulmonary disease (COPD) is characterized by persistent airflow limitation and chronic airway and systemic inflammation. Acute exacerbations of COPD (AECOPD) accelerate symptom deterioration, impair lung function, and are strongly associated with hospitalization, readmission, and increased mortality risk (Hurst et al., 2010; Patel, 2024). Beyond the acute respiratory burden, AECOPD is accompanied by a heightened systemic inflammatory response. During exacerbations, circulating inflammatory mediators such as interleukin-6 (IL-6), interleukin-8 (IL-8), and tumor necrosis factor-α (TNF-α) often rise, and acute-phase responses reflected by high-sensitivity C-reactive protein (hs-CRP) are also frequently observed. These inflammatory changes have been linked to impaired endothelial and metabolic homeostasis and skeletal muscle dysfunction, thereby contributing to activity limitation and a vicious cycle of “inflammation–functional decompensation–recurrent exacerbation” (Gan et al., 2004; Garcia-Rio et al., 2010; Ye et al., 2023). Accordingly, strategies capable of attenuating systemic inflammation while restoring dyspnea-limited functional capacity during the acute and early recovery phases are critical to improving prognosis and reducing readmission risk (Agusti et al., 2016; Patel, 2024).

Pulmonary rehabilitation, with exercise training as a central component, is a cornerstone of comprehensive COPD care and typically integrates aerobic training, resistance training, and respiratory muscle interventions (Wang and Wu, 2024). From an exercise physiology perspective, exercise training can improve skeletal muscle oxidative capacity and ventilatory efficiency, and it may influence systemic inflammation through mechanisms involving myokine-related signaling, autonomic regulation, and ventilation–perfusion interactions under dynamic lung hyperinflation (Pedersen and Febbraio, 2008; O’Donnell et al., 2001; Butler et al., 2020; Wang and Wu, 2024). However, hospitalized AECOPD patients represent a physiologically heterogeneous population. Disease severity, oxygenation reserve, muscle wasting, and comorbidity burden vary substantially across individuals, leading to marked differences in exercise tolerance and safety margins (Agusti et al., 2016; Patel, 2024). Consequently, conventional “one-size-fits-all” inpatient rehabilitation programs often struggle to concurrently optimize safety and efficacy, and high-quality evidence guiding intensity selection and progression in this acute setting remains limited (Puhan et al., 2016; Jenkins et al., 2024).

Exercise intensity is a key determinant of the dose–response relationship, because it directly shapes metabolic load, ventilatory demand, autonomic perturbation, and the overall inflammatory milieu (Karvonen et al., 1957; Wang and Wu, 2024; American College of Sports Medicine, 2025). Although rehabilitation during or after AECOPD has generally been shown to improve symptoms and some inflammatory markers (Thompson et al., 2013; Puhan et al., 2016; Jenkins et al., 2024), the evidence has not consistently demonstrated a reproducible efficacy gradient across different intensity regimens, nor has it established a universal “optimal intensity range.” Physiologically, training that is too mild may not reach the stimulus threshold required for tissue repair and functional restoration, whereas training that is too demanding may increase oxidative stress and inflammatory signaling, producing an overtraining-like response that blunts or counteracts potential benefits (Ribeiro et al., 2022; Magni et al., 2025). For AECOPD, this intensity dilemma becomes particularly relevant because baseline systemic inflammation is elevated, oxygenation reserve is often constrained, and skeletal muscle dysfunction is common (Agusti et al., 2016; Wang and Wu, 2024). Therefore, defining clinically feasible intensity–response patterns and their safety boundaries in hospitalized AECOPD has both physiological and methodological importance.

Graded exercise rehabilitation aims to operationalize individualized prescription by implementing stepped training based on functional baseline and disease stage, with structured monitoring to maximize benefit within a tolerable physiological range (Agusti et al., 2016; Puhan et al., 2016; Wang and Wu, 2024). While theoretical frameworks and emerging studies support graded strategies in AECOPD (Puhan et al., 2016; Jenkins et al., 2024), a key gap persists. It remains unclear whether different intensity levels yield meaningfully different anti-inflammatory and functional effects, and whether such differences follow a stable pattern when intensity is matched to disease severity (Puhan et al., 2016; Jenkins et al., 2024). Addressing this gap would strengthen the evidence base for inpatient intensity selection and progression, and facilitate the development of generalizable rehabilitation pathways (Agusti et al., 2016; Jenkins et al., 2024; Wang and Wu, 2024).

Based on this background, we hypothesized that, during early rehabilitation for hospitalized AECOPD, exercise delivered at different intensity levels would yield discernible differences in anti-inflammatory and functional effects, and that an intensity strategy aligned with patient condition could optimize the balance among inflammation attenuation, functional improvement, and risk control (Thompson et al., 2013; Puhan et al., 2016; Wang and Wu, 2024). To test this hypothesis, we developed a standardized and feasible graded exercise rehabilitation protocol informed by a systematic literature review, expert consultation, and preliminary testing (Garber et al., 2011). Using a prospective, randomized controlled, assessor-blinded design comparing a graded-intensity intervention with conventional rehabilitation, we examined changes in systemic inflammatory biomarkers and functional outcomes. We further explored efficacy differences when exercise intensity was matched to disease severity grades, aiming to support physiologically informed, individualized exercise prescription for hospitalized AECOPD patients (Agusti et al., 2016; Puhan et al., 2016; Jenkins et al., 2024; Wang and Wu, 2024).

## 2 Materials and methods

### 2.1 Study design

This prospective, randomized controlled, assessor-blinded clinical trial was conducted from August 2024 to June 2025 at a tertiary Grade A hospital in Guizhou Province and is reported in accordance with CONSORT guidelines. A total of 150 patients were enrolled, and 141 completed the trial and were included in the final analysis after withdrawals. At admission, participants were assigned to a prespecified severity grade (I–III) before randomization using the criteria in **Table 1**.

**Table 1:** GOLD-defined severity grading criteria (Grades I-III) for AECOPD used in this trial.

Grade	Respiratory pattern	Oxygenation requirement	Ventilatory status	Mental status/effort
I	RR ≤ 24 breaths/min; HR < 95 beats/min	Hypoxemia improves with inhaled oxygen concentration 24–35%	No increase in PaCO_2_	No accessory muscle use; no change in mental status
II	RR > 24 breaths/min	Hypoxemia improves with inhaled oxygen concentration > 35%	Hypercapnia present: PaCO_2_ rises from baseline or reaches 50–60 mmHg	Accessory muscle use; mental status unchanged
III	RR > 24 breaths/min	Hypoxemia requires inhaled oxygen concentration > 40% to improve	Hypercapnia with PaCO_2_ rising from baseline or > 60 mmHg, and/or acidosis with pH ≤ 7.25	Accessory muscle use; acute mental status change but able to cooperate with treatment

The grading criteria were prespecified before recruitment and applied at enrollment for stratified randomization; they reflect acute respiratory pattern, oxygen requirement, ventilatory status, and mental status consistent with GOLD 2024 recommendations.

### 2.2 Sample size calculation

The sample size was calculated based on IL-6 as the primary outcome. Assuming a two-sided α of 0.05, a statistical power of 90%, and an expected between-group difference in IL-6 change derived from preliminary data, the minimum required sample size was estimated to be 66 participants per group. Allowing for an anticipated attrition rate of 15%, the planned total sample size was set at 150 participants. After accounting for withdrawals, 141 participants completed the trial, yielding 70–71 participants per group, which exceeded the minimum required sample size.

### 2.3 Randomization, allocation concealment, and blinding

Randomization used a stratified block procedure by severity grade (I–III) with a 1:1 allocation to the Study group (graded exercise rehabilitation) or the Control group (conventional exercise rehabilitation). The final allocation within each severity stratum was as follows: Control group—Grade I (n=24), Grade II (n=24), and Grade III (n=23); Study group—Grade I (n=23), Grade II (n=24), and Grade III (n=23). This distribution confirmed that stratification achieved comparable subgroup sizes across groups. An independent statistician generated the randomization sequence using computer software, and allocation concealment was ensured through centralized randomization. Due to the nature of the intervention, rehabilitation therapists were not blinded; however, outcome assessors and data analysts remained blinded to group assignment, and masking was maintained throughout assessment and analysis.

### 2.4 Severity grade classification

Severity Grades I–III were assigned using an existing AECOPD severity grading framework described in the GOLD 2024 report (Patel, 2024), rather than a study-developed system. Grading was performed at enrollment before randomization according to these prespecified criteria (**Table 1**).

### 2.5 Participants

#### 2.5.1 Eligibility criteria

Inclusion criteria were age 40–80 years; a confirmed diagnosis of COPD based on the 2024 GOLD criteria; hospitalization for AECOPD; adequate communication ability to complete assessments; and no participation in a structured exercise rehabilitation program within the preceding 3 months. Exclusion criteria were severe cardiovascular disease, acute bronchial asthma, pulmonary embolism, pneumothorax, or hemoptysis; active malignancy; neuromuscular or musculoskeletal disorders affecting mobility; immunodeficiency; or poor expected adherence.

#### 2.5.2 Recruitment and baseline procedures

Screening was completed within 24 h of admission. Eligible patients underwent baseline assessments, provided written informed consent, were graded by severity, and were then randomized.

### 2.6 Intervention methods

#### 2.6.1 Standard medical care (both groups)

All participants received standard inpatient medical treatment for AECOPD in accordance with routine clinical practice, including bronchodilator therapy, systemic glucocorticoids, oxygen therapy as indicated, anti-infective treatment when clinically required, airway clearance support, and nutritional support where appropriate. Pharmacologic treatment was directed by the treating respiratory physicians according to routine guideline-based inpatient care and was not protocolized by the trial. Because both groups were managed within the same clinical setting after randomization, major systematic between-group differences in treatment approach were not expected; however, detailed medication exposure was not prespecified as an analytic covariate.

#### 2.6.2 Control group: conventional exercise rehabilitation

In addition to standard medical care, the control group received conventional exercise rehabilitation consisting of breathing retraining and basic functional exercise. Breathing retraining included pursed-lip breathing and diaphragmatic breathing, performed twice daily for 5–10 min per session. Functional training focused on activities of daily living and simple upper- and lower-limb exercises, delivered twice daily, with each exercise performed for 2–3 sets of 8–12 repetitions. Walking training was introduced progressively as clinical stability allowed.

#### 2.6.3 Study group: severity-adapted graded exercise rehabilitation

In addition to standard medical care, the study group received a severity-adapted graded exercise rehabilitation program delivered twice daily from admission until discharge. The program applied an inverse severity–intensity matching strategy, whereby patients with more severe disease received lower-intensity exercise, those with moderate severity received moderate-intensity exercise, and those with milder disease received higher-intensity exercise.

#### 2.6.4 Exercise intensity prescription and titration

Target heart rate (THR) was prescribed using the Karvonen heart-rate-reserve (HRR) method (THR = HR_rest + %HRR × [HR_max − HR_rest]), with HR_max estimated as 220 − age (Meeusen et al., 2013; Yang et al., 2024). The intended training range was 60%–80% HRR; however, in AECOPD the HRR range served as an upper reference rather than a rigid target because chronotropic responses may be altered by acute respiratory distress and concomitant medications. Intensity was individualized and titrated using symptom- and safety-limited criteria, including Borg dyspnea score (target 4–6), oxygenation stability, and hemodynamic safety. Training load/volume was increased when Borg was <4 and reduced when Borg exceeded 6.

#### 2.6.5 Progression and stopping criteria

Intensity progression and within-session adjustments were guided by prespecified objective physiological markers rather than subjective tolerance alone. Progression was permitted only when patients were clinically stable and met initiation criteria (e.g., SpO_2_ ≥90% and blood pressure ≥90/60 mmHg) and the preceding session was completed without triggering suspension criteria. Sessions were down-titrated or suspended for predefined intolerance, including desaturation (SpO_2_ <85% for >3 min), respiratory instability (RR <5 or >30 breaths/min, or persistent dyspnea for >3 min), hemodynamic instability (BP >180/110 mmHg or <90/60 mmHg; MAP <65 mmHg; or ≥20% change from baseline BP lasting >3 min), cardiac events (arrhythmia, acute heart failure, acute myocardial infarction, or HR exceeding the upper THR limit for >3 min), or symptoms such as chest tightness/pain, dizziness, profuse sweating, pallor, or inability/refusal to continue. Detailed monitoring, progression rules, and suspension criteria are provided in **Supplementary Table 1**, and standardized emergency management procedures are described in **Supplementary Appendix S1**. Each session duration (30–45 min) refers to the planned core rehabilitation time delivered in an intermittent format with rest as needed; therefore, sessions were not continuous high-load exercise, and the actual work-to-rest ratio was individualized according to symptom- and safety-limited criteria (**Supplementary Table 1**). Warm-up and cool-down (5–10 min each) were prescribed when feasible.

#### 2.6.6 Emergency management during rehabilitation sessions

Standardized procedures were implemented to ensure safety during in-hospital rehabilitation. Any suspected intolerance prompted immediate cessation of exercise, rapid clinical assessment (airway, breathing, circulation, neurological status), and adjustment of oxygen support as needed, with timely physician notification and escalation according to institutional pathways. Events were documented and rehabilitation was resumed only after clinical stabilization using a conservative re-initiation strategy (**Appendix S1**).

### 2.7 Outcome measures

#### 2.7.1 Primary outcomes

The primary outcomes were changes in systemic inflammatory biomarkers and acute-phase reactants, including IL-6, IL-8, TNF-α, hs-CRP, and WBC.

#### 2.7.2 Blood sampling and laboratory assays

Fasting venous blood samples were collected in the morning at each assessment time point. Briefly, 3 mL of blood was drawn from an antecubital vein into a clot-activator tube, centrifuged at 3,000 rpm at 4 °C for 10 min, and serum was separated, aliquoted, and stored at −80 °C until analysis. To minimize inter-assay variability, serum samples were analyzed in a single batch. Serum IL-6, IL-8, and TNF-α were quantified using double-antibody sandwich enzyme-linked immunosorbent assays (ELISA) with an automated microplate reader (Tecan Sunrise, Switzerland). Hs-CRP was measured using a commercially available kit according to the manufacturer’s quality control and calibration procedures. WBC was determined by the hospital clinical laboratory using the same automated hematology analyzer under routine internal quality control.

#### 2.7.3 Secondary outcomes

Functional capacity: Functional exercise tolerance was assessed using the 6-minute walk test (6MWT) in accordance with ATS/ERS recommendations. Standardized instructions were provided, and the total distance walked in 6 min was recorded. Oxygen flow was kept constant for participants receiving supplemental oxygen. For safety, SpO_2_ and heart rate were monitored continuously throughout the test using a portable pulse oximeter. The test was terminated if prespecified safety criteria were met (e.g., severe desaturation, excessive dyspnea, chest symptoms, dizziness, or patient refusal), consistent with the suspension criteria described in **Supplementary Table 1**.

Dyspnea: Dyspnea severity was evaluated using the modified British Medical Research Council (mMRC) scale (Bestall et al., 1999; Singh et al., 2014).

Health status: Health-related symptom burden was assessed with the COPD Assessment Test (CAT) (Jones et al., 2009; Kon et al., 2014).

Psychological status: Anxiety and depressive symptoms were assessed using the Hospital Anxiety and Depression Scale (HADS), including HADS-A and HADS-D subscales (Zigmond and Snaith, 1983; Puhan et al., 2008).

### 2.8 Data collection and management

#### 2.8.1 Data collection

All outcomes were assessed at two time points: baseline (within 24 h of admission) and post-intervention (within 24 h after completing the intervention, when feasible). Assessments were performed by trained assessors blinded to group allocation using standardized procedures. For questionnaire-based measures (mMRC, CAT, HADS), validated Chinese versions were administered face-to-face and checked onsite for completeness.

Clinical variables, including non-invasive ventilation parameters and complications during hospitalization, were extracted from physician orders and respiratory therapy records and cross-verified against electronic medical records. Length of stay was obtained from the electronic medical record system.

#### 2.8.2 Data entry and quality control

Data were recorded using case report forms and entered into an electronic database after de-identification. Double data entry was performed by two independent personnel, and discrepancies were resolved by referring to source documents with an auditable record of changes. Laboratory results were imported in batches with documentation of assay batch information; where appropriate, assay batch was considered in sensitivity analyses. Range checks and logic checks were applied during data entry. The final de-identified dataset was locked by an independent data manager after completion of data cleaning and approval of the statistical analysis plan.

#### 2.8.3 Feasibility and tolerance outcomes

Feasibility outcomes were prespecified and extracted from standardized rehabilitation logs. For each participant, planned sessions were defined as the number of sessions scheduled during hospitalization (two sessions per day, capped at 14 days or until discharge). Completed sessions were those finished as scheduled. A session was classified as down-titrated if intensity or volume was reduced (e.g., lower target HRR, shorter work intervals, or longer rest) in response to prespecified tolerance/safety criteria, and terminated early if the session was stopped before completion. Down-titration and early termination were summarized at the session level, and percentages were calculated using the total number of delivered sessions within each subgroup as the denominator. Reasons for adjustment were categorized (e.g., transient desaturation, excessive dyspnea, chest symptoms, musculoskeletal discomfort) according to **Supplementary Table 1** and **Supplementary Appendix S1**. In this symptom-limited inpatient setting, actual delivered rehabilitation dose was operationalized using session-based metrics, including planned sessions, completed sessions, completion rate, session-level down-titration, and early termination.

### 2.9 Statistical analysis

Statistical analyses were conducted by a statistician blinded to group allocation using SPSS version 29.0. Continuous variables were assessed for normality using the Shapiro–Wilk test and for homogeneity of variance using Levene’s test. Normally distributed data are reported as mean ± standard deviation, and non-normally distributed data as median (interquartile range). Categorical variables are summarized as number (percentage).

Within-group pre–post changes were examined using paired t-tests or Wilcoxon signed-rank tests, as appropriate. The primary between-group comparison focused on post-intervention outcomes between groups and was tested using independent-samples t tests or Mann–Whitney U tests, depending on distribution. For sensitivity analyses of primary continuous outcomes, linear mixed-effects models were fitted with group, time, and group×time interaction as fixed effects; baseline value of the outcome and relevant covariates (including assay batch when applicable) were included when appropriate.

Between-group comparisons of categorical variables were performed using the χ² test or Fisher’s exact test. For prespecified subgroup analyses, pairwise *post-hoc* comparisons were conducted only when the omnibus test was significant. A two-sided α of 0.05 was considered statistically significant. Because five inflammatory biomarkers (IL-6, IL-8, TNF-α, hs-CRP, and WBC) were prespecified as primary outcomes within the same biomarker family, multiplicity was additionally considered using a Bonferroni-adjusted significance threshold of P < 0.01 (0.05/5) as a conservative sensitivity framework. Findings that did not meet this adjusted threshold were interpreted as supportive rather than definitive.

To evaluate intensity-stratified responses under the severity-adapted prescription, post-intervention outcome levels were compared across the three prespecified severity strata (Severity Grade I–III) within each group using one-way ANOVA for approximately normally distributed variables (or Kruskal–Wallis tests for non-normally distributed variables). For ANOVA-based comparisons, the omnibus test statistic is reported as the F value (with its corresponding P value). When the omnibus test was significant, pairwise *post-hoc* comparisons were performed using the least significant difference (LSD) method. For the Study group, Severity Grade I–III strata corresponded to different prescribed exercise intensity grades via inverse severity–intensity matching, whereas strata in the Control group reflected baseline severity only.

## 3 Results

### 3.1 Participant flow and baseline characteristics

**Figure 1:**
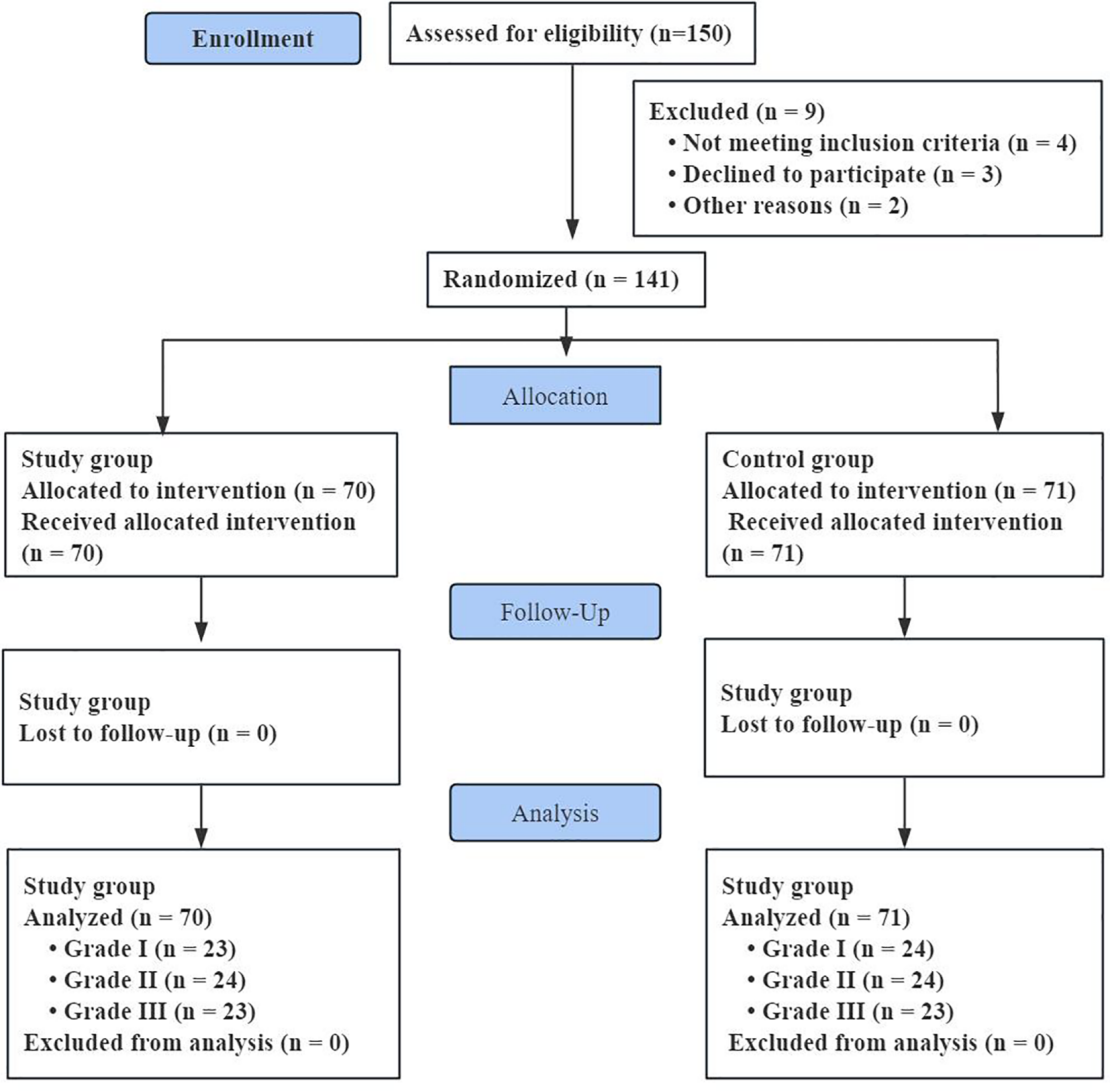
CONSORT flow diagram of participant enrollment, allocation, follow-up, and analysis.

Participant flow is summarized in **Figure 1**. A total of 150 hospitalized patients with AECOPD were screened and randomized. During follow-up, 9 participants (6.0%) were excluded from the final analysis because of transfer to another hospital, withdrawal, or incomplete outcome data (Study group: n=5; Control group: n=4). Ultimately, 141 participants completed both baseline and post-intervention assessments and were included in the analysis (Study group, n=70; Control group, n=71). Baseline demographic characteristics were similar between groups (all P > 0.05; **Table 2**). Baseline inflammatory and functional measures stratified by severity grade are shown in **Table 3**. The primary analysis was conducted using complete cases with both baseline and post-intervention assessments.

**Table 2:** Baseline characteristics of the study participants.

Variable	Category	Study group	Control group	Statistic	P
Age (years)		69.13 ± 7.14	67.99 ± 9.00	t=0.836	0.405
Sex	Male, n (%)	52 (74.3)	50 (70.4)	χ²=0.263	0.608
BMI (kg/m²)		22.40 ± 2.88	23.23 ± 3.70	t=-1.478	0.142
Duration of COPD (years)		9.17 ± 6.55	9.13 ± 9.50	t=0.025	0.98
Hospitalizations for Acute Exacerbation (past year)		2.00[1.00,2.00]	2.00[1.00,2.00]	Z=0.466	0.617
Smoking History	Yes, n (%)	51(72.9)	51(71.8)	χ²=0.019	0.892
Daily Cigarette Consumption (sticks)		30.00[0.00,40.00]	25.00[0.00,35.00]	Z=0.359	0.717
Smoking Duration (years)		10.00[0.00,20.00]	15.00[0.00,20.00]	Z=0.586	0.552
Dust/Soot Exposure History	Yes, n (%)	26(37.1)	29(40.8)	χ²=0.203	0.652
Duration of Dust-Exposure Work (years)		8.57 ± 12.63	8.82 ± 12.39	t=-0.117	0.907
Occupation	Worker/Peasant, n (%)	57(81.4)	60(84.5)	Fisher¹	0.944
	Clerical, n (%)	3(4.3)	2(2.8)		
	Freelance, n (%)	5(7.1)	4(5.6)		
	Unemployed, n (%)	5(7.1)	5(7.0)		
Education Level	Primary school or below, n (%)	47(67.1)	47(66.2)	Fisher¹	0.534
	Junior high school, n (%)	16(22.9)	20(28.2)		
	Technical secondary school or above, n (%)	7(10.0)	4(5.6)		
Marital Status	Married, n (%)	47(67.1)	52(73.2)	Fisher¹	0.311
	Unmarried, n (%)	2(2.9)	1(1.4)		
	Divorced, n (%)	3(4.3)	0(0)		
	Widowed, n (%)	18(25.7)	18(25.4)		
Residence	Urban, n (%)	14(20.3)	14(19.7)	χ²=1.029	0.598
	Rural, n (%)	55(79.7)	57(80.3)		
Living Situation	With spouse, n (%)	29(41.4)	32(45.1)	χ²=2.389	0.303
	With children, n (%)	35(50.0)	28(39.4)		
	Living alone, n (%)	6(8.6)	11(15.5)		
Monthly Household Income Per Capita (CNY)	<3000, n (%)	47(67.1)	49(69.0)	Fisher¹	0.589
	3000-5000, n (%)	20(28.6)	21(29.6)		
	≥5000, n (%)	3(4.3)	1(1.4)		
Medical Payment Method	Employee Health Insurance, n (%)	9(12.9)	7(9.9)	Fisher¹	0.504
	Resident Health Insurance, n (%)	60(85.7)	64(90.1)		
	Self-payment, n (%)	1(1.4)	0(0.0)		
Home Oxygen Therapy	Yes, n (%)	17(24.3)	25(35.2)	χ²=2.012	0.156
Prior Rehabilitation Training	Yes, n (%)	17(24.3)	22(31.0)	χ²=0.791	0.374

Continuous variables are presented as mean ± SD or median (IQR) and were compared using independent-samples t tests or Mann–Whitney U tests, as appropriate. Categorical variables are presented as n (%) and were compared using the χ² test or Fisher’s exact test. For binary variables, only one level is shown to reduce redundancy; the complementary category is implicit.

**Table 3:** Baseline inflammatory and functional status by group within each severity grade.

Variable (baseline)	Grade I study (n=23)	Grade I control (n=24)	Grade I statistic (p)	Grade II study (n=24)	Grade II control (n=24)	Grade II statistic (p)	Grade III study (n=23)	Grade III control (n=23)	Grade III statistic (p)
IL-6 (pg/mL)	35.03 ± 7.98	33.43 ± 9.13	0.69 (0.497)	35.97 ± 10.15	36.88 ± 6.63	-0.39 (0.699)	36.52 ± 10.92	34.85 ± 10.42	0.53 (0.601)
IL-8 (pg/mL)	83.80 ± 22.94	79.71 ± 31.91	0.53 (0.598)	88.37 ± 30.83	85.21 ± 31.78	0.37 (0.713)	92.02 ± 37.12	95.49 ± 35.19	-0.33 (0.746)
TNF-α (pg/mL)	59.82 ± 16.04	57.51 ± 13.68	0.56 (0.580)	58.18 ± 13.81	57.80 ± 14.18	0.10 (0.919)	58.24 ± 12.43	60.27 ± 15.62	-0.48 (0.633)
WBC (×10^9^/L)	8.95 ± 4.12	9.29 ± 3.01	-0.33 (0.741)	10.09 ± 4.45	10.71 ± 5.57	-0.43 (0.671)	10.37 ± 4.63	10.36 ± 4.73	0.01 (0.992)
hs-CRP (mg/L)	13.59 ± 13.01	13.21 ± 9.80	0.11 (0.914)	18.50 ± 12.16	16.13 ± 11.76	0.71 (0.484)	17.41 ± 11.91	17.68 ± 10.33	-0.08 (0.938)
6MWT distance (m)	277.04 ± 47.87	271.29 ± 67.35	0.34 (0.738)	256.22 ± 39.07	256.21 ± 52.16	0.00 (0.999)	252.09 ± 47.47	242.96 ± 69.69	0.53 (0.599)
mMRC (score)	3.00 [3.00, 4.00]	3.50 [3.00, 4.00]	245.0 (0.483)	4.00 [3.00, 4.00]	4.00 [3.00, 4.00]	275.5 (0.964)	4.00 [4.00, 4.00]	4.00 [3.00, 5.00]	226.0 (0.643)
CAT (score)	25.96 ± 3.49	26.29 ± 3.91	-0.32 (0.752)	26.78 ± 3.93	26.58 ± 4.67	0.16 (0.877)	27.48 ± 3.62	28.00 ± 4.13	-0.46 (0.645)
HADS-A (score)	9.00 [8.00, 10.00]	10.00 [9.00, 12.00]	184.0 (0.067)	9.00 [8.00, 11.00]	10.00 [8.00, 11.00]	233.0 (0.401)	10.00 [8.00, 12.00]	10.00 [8.00, 12.00]	254.0 (0.891)
HADS-D (score)	9.00 [8.00, 10.00]	9.50 [8.00, 11.00]	246.5 (0.501)	9.00 [7.00, 11.00]	9.00 [8.00, 10.00]	258.5 (0.648)	10.00 [8.00, 12.00]	9.00 [8.00, 11.00]	241.0 (0.777)

Data are mean ± SD for approximately normally distributed variables and median [IQR] for ordinal/non-normally distributed variables. Within each severity grade, between-group comparisons used independent-samples t tests or Mann–Whitney U tests as appropriate; the reported statistic corresponds to the test applied (t or U), followed by the p value.

Baseline inflammatory biomarkers and functional status were further examined within each prespecified severity grade to support interpretation of the severity-stratified (inverse matching) analyses. As shown in **Table 3**, baseline values were broadly comparable between groups within Grades I–III across systemic inflammatory markers (IL-6, IL-8, TNF-α, WBC, hs-CRP) and functional outcomes (6MWT distance, mMRC, Borg dyspnea, CAT, HADS-A, HADS-D, and ADL), with no statistically significant between-group differences within any severity grade (all P > 0.05).

### 3.2 Intervention feasibility and tolerance

Actual delivered intervention dose is summarized in **Table 4** using session-based rehabilitation delivery metrics. Overall, completion of planned sessions was acceptable across severity grades, with lower completion and higher rates of down-titration/early termination observed in Grade III compared with Grades I–II. In Grade III, the completion rate was 81% in the Study group versus 75% in the Control group, and down-titration occurred in 13.0% versus 9.6% of sessions, respectively. Early termination occurred in 4.3% of sessions in the Study group and 3.0% in the Control group. The most common reasons for adjustment were desaturation and dyspnea. No serious adverse events related to rehabilitation were recorded. These session-based indicators characterize the actual delivered dose of the twice-daily inpatient rehabilitation program and show that individualized dose adjustment was frequently feasible, particularly in Grade III.

**Table 4:** Actual delivered rehabilitation dose, feasibility, and tolerance by group and severity grade.

Group	Severity grade	n	Planned sessions, median (IQR)	Completed sessions, median (IQR)	Completion rate (%)	Sessions requiring down-titration, n (%)	Sessions terminated early, n (%)	Most common reason(s) for adjustment (top 2)
Control	Grade I	24	18 (14–22)	16 (13–20)	88	8 (3.2)	2 (0.8)	fatigue; dyspnea
	Grade II	24	18 (15–22)	15 (12–19)	83	14 (5.8)	4 (1.7)	dyspnea; desaturation
	Grade III	23	16 (12–20)	12 (9–16)	75	22 (9.6)	7 (3.0)	desaturation; dyspnea
Study	Grade I	23	18 (14–22)	17 (14–21)	92	10 (4.3)	3 (1.3)	dyspnea; fatigue
	Grade II	24	18 (15–22)	16 (13–20)	87	18 (7.5)	5 (2.1)	dyspnea; desaturation
	Grade III	23	16 (12–20)	13 (10–17)	81	30 (13.0)	10 (4.3)	desaturation; dyspnea

Down-titration and early termination are reported at the session level (number and percentage of all delivered sessions within each subgroup). Percentages were calculated using the total number of delivered sessions within each subgroup as the denominator.

### 3.3 Feasibility and safety of the 6MWT

Completion versus abortion rates of the 6MWT by group and severity grade are summarized in **Table 5**. Overall, completion was high in Grades I–II in both groups (≥91.7%). In Grade III, the 6MWT was completed by 18/23 (78.3%) participants in the Control group and 19/23 (82.6%) in the Study group, while abortion occurred in 5/23 (21.7%) and 4/23 (17.4%), respectively. The most common reasons for abortion were desaturation and dyspnea. For safety, SpO_2_ and heart rate were monitored continuously during testing.

**Table 5:** Feasibility of the 6-minute walk test (6MWT) by group and severity grade.

Group	Severity grade	n	6MWT completed, n (%)	6MWT aborted, n (%)	Main reason for abortion (top 1–2)
Control	Grade I	24	23 (95.8)	1 (4.2)	dyspnea/desaturation
	Grade II	24	22 (91.7)	2 (8.3)	dyspnea/desaturation
	Grade III	23	18 (78.3)	5 (21.7)	desaturation/dyspnea
Study	Grade I	23	22 (95.7)	1 (4.3)	dyspnea/desaturation
	Grade II	24	22 (91.7)	2 (8.3)	dyspnea/desaturation
	Grade III	23	19 (82.6)	4 (17.4)	desaturation/dyspnea

Abortion indicates premature termination of the 6MWT due to prespecified safety criteria (**Supplementary Table 1**).

### 3.4 Overall effects of graded exercise rehabilitation

#### 3.4.1 Primary outcomes: systemic inflammatory markers

**Figure 2:**
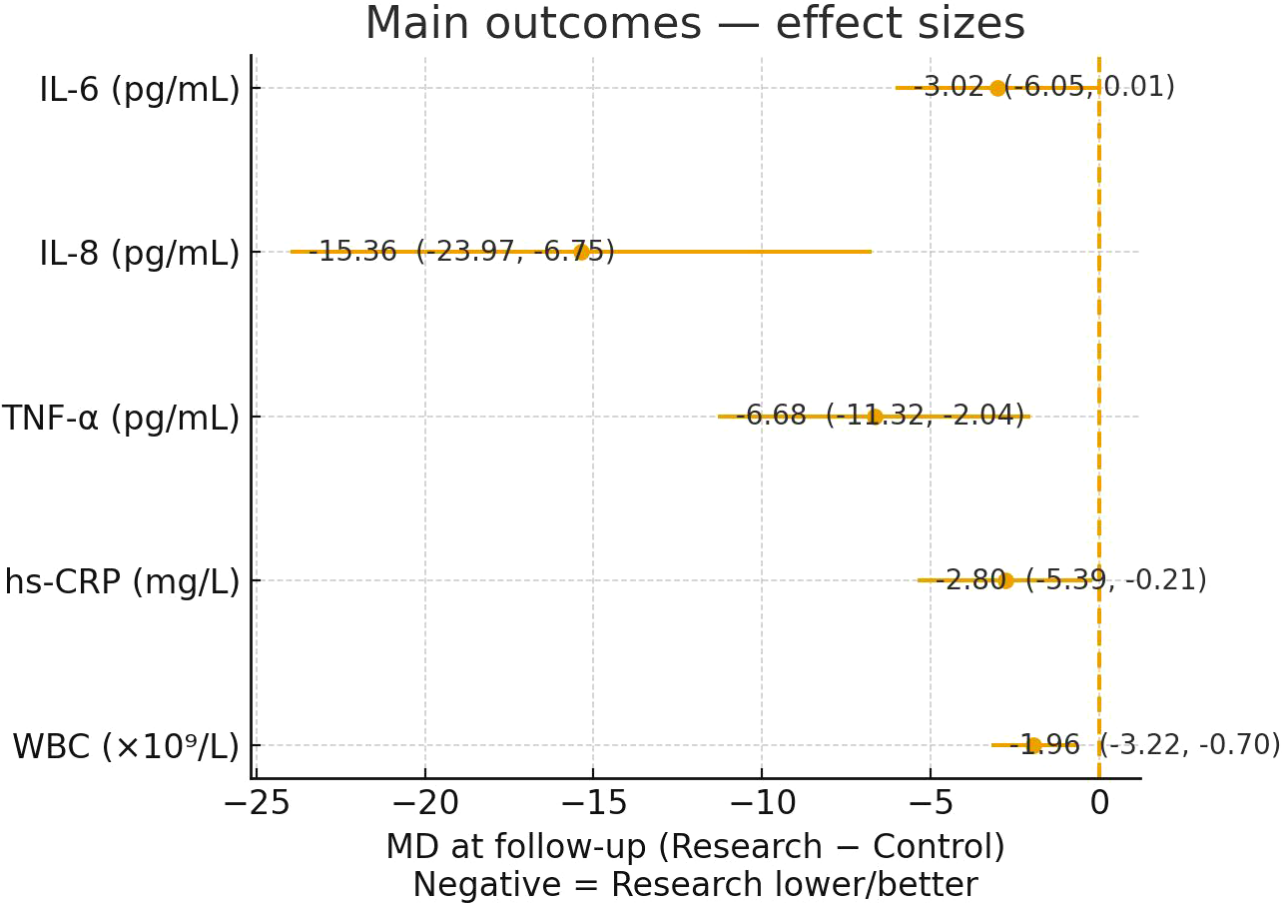
Main outcomes—between-group effect sizes at post-intervention. Mean difference (MD) at follow-up is calculated as Study − Control and shown with 95% CI; the vertical dashed line indicates MD = 0. Negative values indicate lower (better) levels in the Study group.

**Figure 3:**
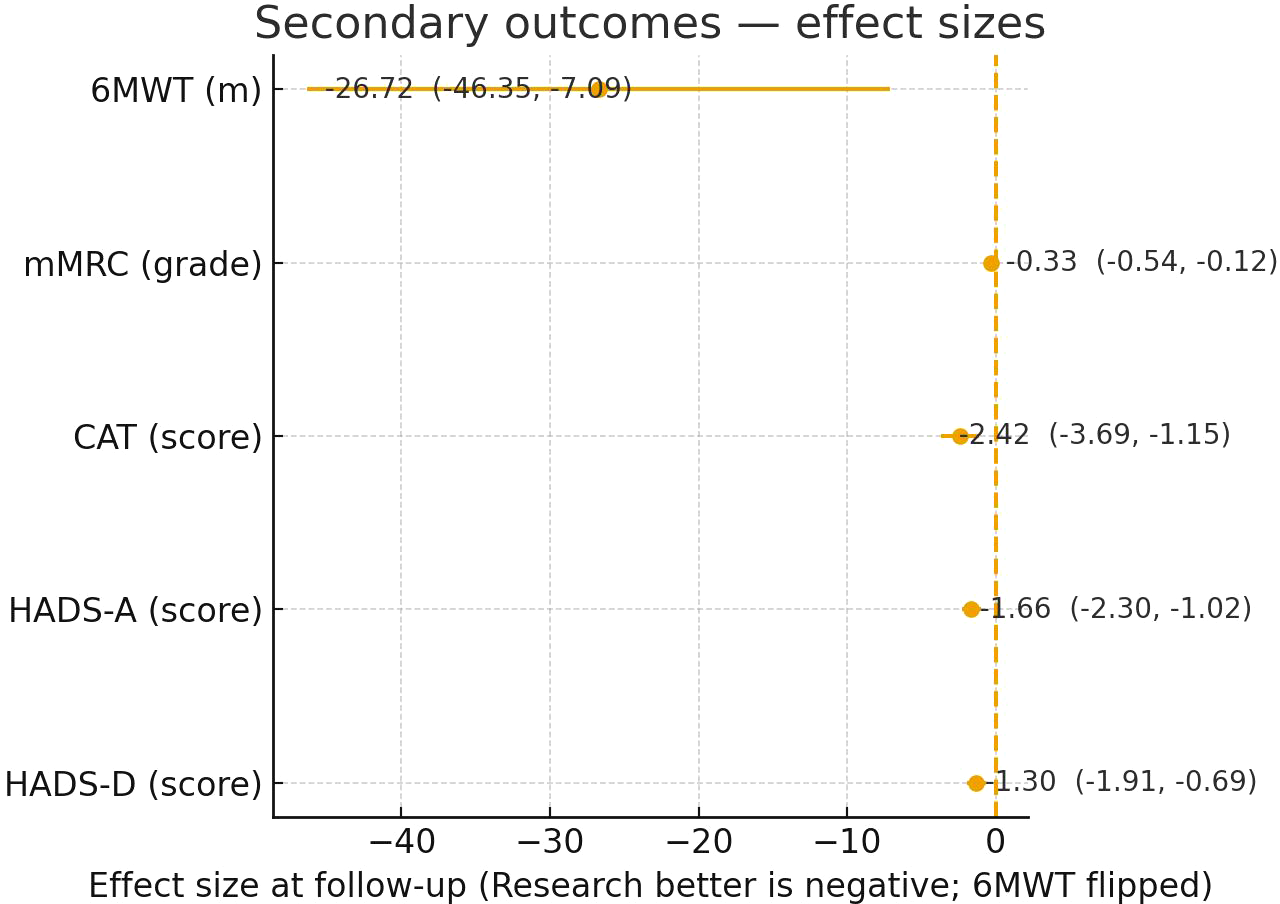
Secondary outcomes—between-group differences at post-intervention. Mean differences are shown with 95% CIs and the dashed line indicates no difference. For mMRC, CAT, HADS-A and HADS-D, MD = Study − Control (values < 0 indicate better status in the Study group). For 6MWT, the value is plotted as Control − Study so that values < 0 consistently indicate “Study better”; the natural-direction difference (Study − Control, meters) is reported in **Table 7**.

After the intervention period, inflammatory markers decreased in both groups. Between-group comparisons at post-intervention indicated more favorable profiles in the Study group for IL-6, IL-8, TNF-α, hs-CRP, and WBC under nominal testing (**Table 6**). Under the conservative Bonferroni-adjusted threshold (P < 0.01) for the five primary inflammatory biomarkers, the between-group differences remained robust for IL-8, TNF-α, and WBC, whereas the differences for IL-6 and hs-CRP were directionally consistent but should be interpreted as supportive findings. **Figure 2** displays the between-group mean difference (Study − Control) at post-intervention with 95% confidence intervals; values < 0 indicate lower levels in the Study group. For **Figures 2**, **3**, effects are shown with a common visual direction in which values < 0 indicate “Study better.” Accordingly, for 6MWT (where higher values indicate improvement), the plotted value is shown as Control − Study, while **Table 7** reports the natural-direction difference (Study − Control) in meters.

**Table 6:** Comparison of primary outcome measures between study and control groups before and after intervention.

Indicator	Group	Before intervention	t	p	After intervention	t	p
IL-6 (pg/mL)	Study Group	35.61 ± 9.47	0.273	0.785	25.18 ± 9.10*	-2.079	0.04
	Control Group	35.22 ± 8.30			28.20 ± 9.26*		
IL-8 (pg/mL)	Study Group	87.14 ± 27.13	0.712	0.478	65.16 ± 28.71*	-3.198	0.002
	Control Group	80.63 ± 31.80			80.52 ± 23.09		
TNF-α (pg/mL)	Study Group	59.40 ± 13.66	-0.201	0.841	48.25 ± 13.33*	-2.635	0.009
	Control Group	58.97 ± 13.71			54.93 ± 14.73		
hs-CRP (mg/L)	Study Group	16.88 ± 11.52	0.213	0.831	9.95 ± 6.92*	-2.056	0.042
	Control Group	16.78 ± 9.96			12.75 ± 8.71*		
WBC (×10^9^/L)	Study Group	9.72 ± 3.97	0.224	0.823	7.12 ± 3.64*	-2.968	0.004
	Control Group	9.69 ± 3.99			9.08 ± 3.98		

*Indicates a statistically significant difference (P < 0.05) in the within-group pre-post paired comparison.

**Table 7:** Comparison of secondary outcome measures between study and control groups before and after intervention.

Indicator	Group	Before intervention	t	p	After intervention	t	p
6MWT(m)	Study Group	262.96 ± 43.42	0.619	0.537	340.30 ± 59.92*	2.668	0.009
	Control Group	257.48 ± 60.26			313.58 ± 59.02*		
mMRC(score)	Study Group	3.54 ± 0.72	-0.484	0.629	1.54 ± 0.58*	-3.057	0.003
	Control Group	3.61 ± 0.82			1.87 ± 0.70*		
CAT(score)	Study Group	26.46 ± 2.87	-0.918	0.36	20.24 ± 3.81*	-3.721	<0.001
	Control Group	26.97 ± 3.73			22.66 ± 3.91*		
HADS-A (score)	Study Group	9.64 ± 2.30	-1.319	0.189	5.09 ± 1.99*	-5.093	<0.001
	Control Group	10.11 ± 1.91			6.75 ± 1.88*		
HADS-D (score)	Study Group	9.46 ± 2.13	-0.445	0.657	4.56 ± 1.80*	-4.213	<0.001
	Control Group	9.61 ± 1.82			5.86 ± 1.87*		

*Indicates a statistically significant difference (P < 0.05) in the within-group pre-post paired comparison.

#### 3.4.2 Secondary outcomes: exercise tolerance, dyspnea, quality of life, and psychological status

Clinical outcomes improved in both groups after the intervention. Between-group comparisons at post-intervention favored the Study group for 6MWT, mMRC, CAT, HADS-A, and HADS-D (**Table 7**). **Figure 3** summarizes the between-group mean differences at post-intervention for secondary outcomes.

#### 3.4.3 Clinical relevance (MCID) of secondary outcomes

In addition to statistical significance, the observed improvements were clinically meaningful. The mean increase in 6MWT exceeded commonly used MCID thresholds in COPD (about 25–33 m) in both groups, with a larger gain in the Study group (≈77 m vs ≈56 m) (**Table 7**) (Singh et al., 2014). The reduction in CAT score exceeded the proposed MCID of 2 points in both groups (≈6.2 vs ≈4.3 points) (Kon et al., 2014). HADS-A and HADS-D also improved beyond the commonly used MCID threshold of ~1.5 points (Study: ≈4.6 and ≈4.9; Control: ≈3.4 and ≈3.8 points) (Puhan et al., 2008). For dyspnea, although a universally accepted MCID for mMRC is not established, a 1-grade reduction is widely considered clinically relevant in pulmonary rehabilitation contexts; both groups improved by more than 1 grade (≈2.0 vs ≈1.7) (**Table 7**) (Spruit et al., 2013).

### 3.5 Intensity-stratified responses under severity-adapted matching

**Figure 4:**
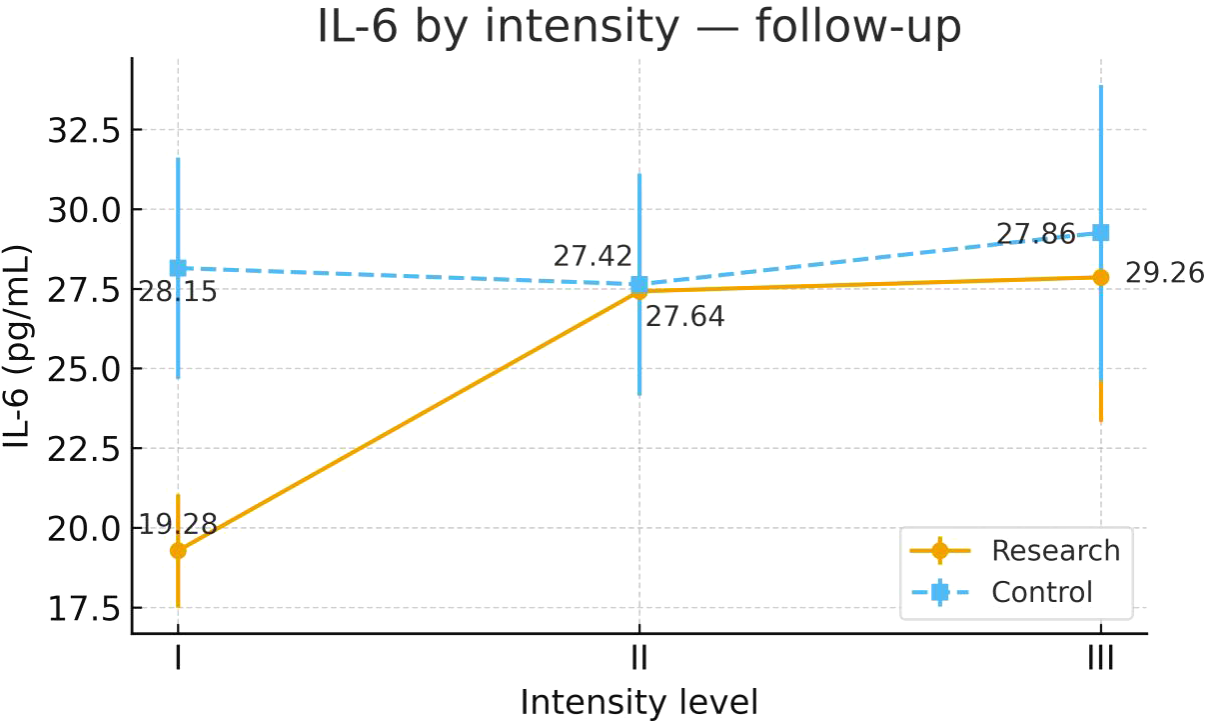
IL-6 after intervention across severity strata.

**Figure 5:**
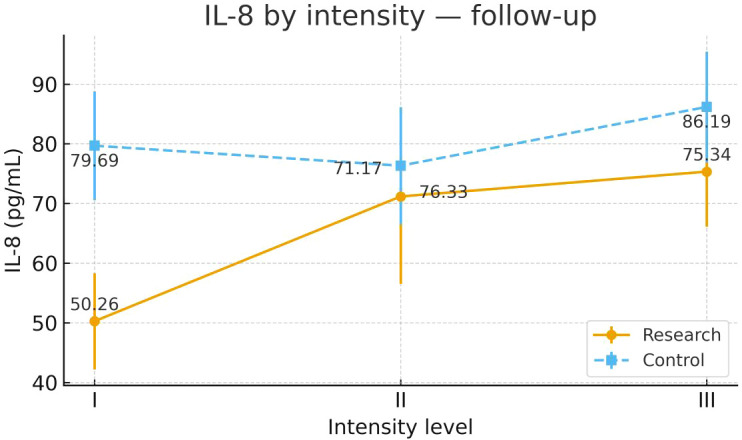
IL-8 after intervention across severity strata.

**Figure 6:**
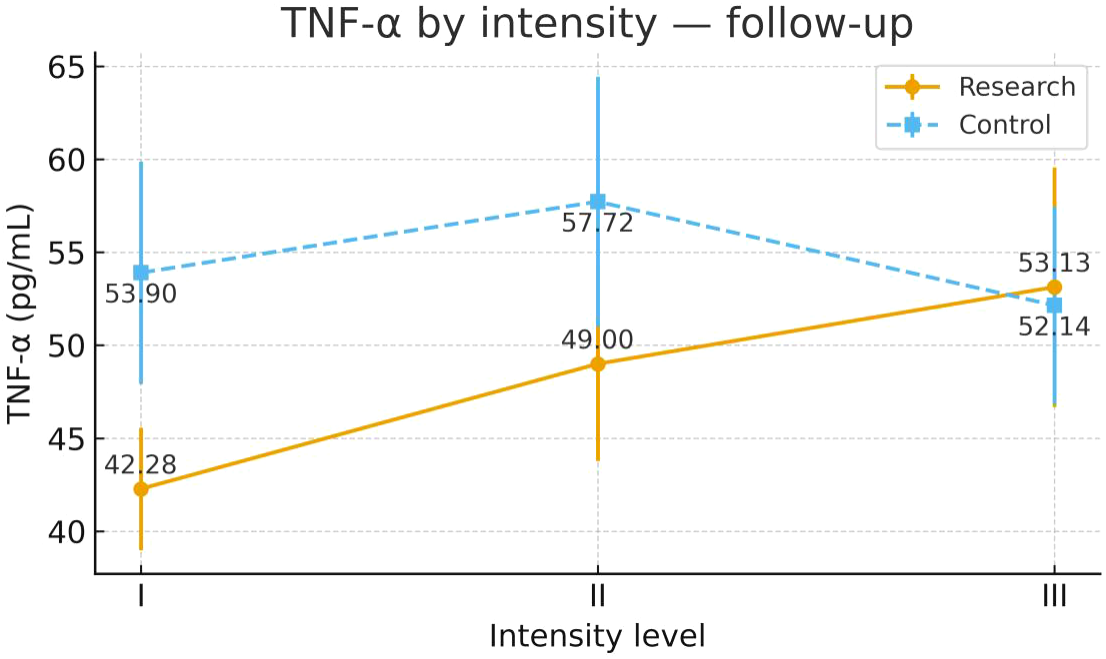
TNF-α after intervention across severity strata.

**Figure 7:**
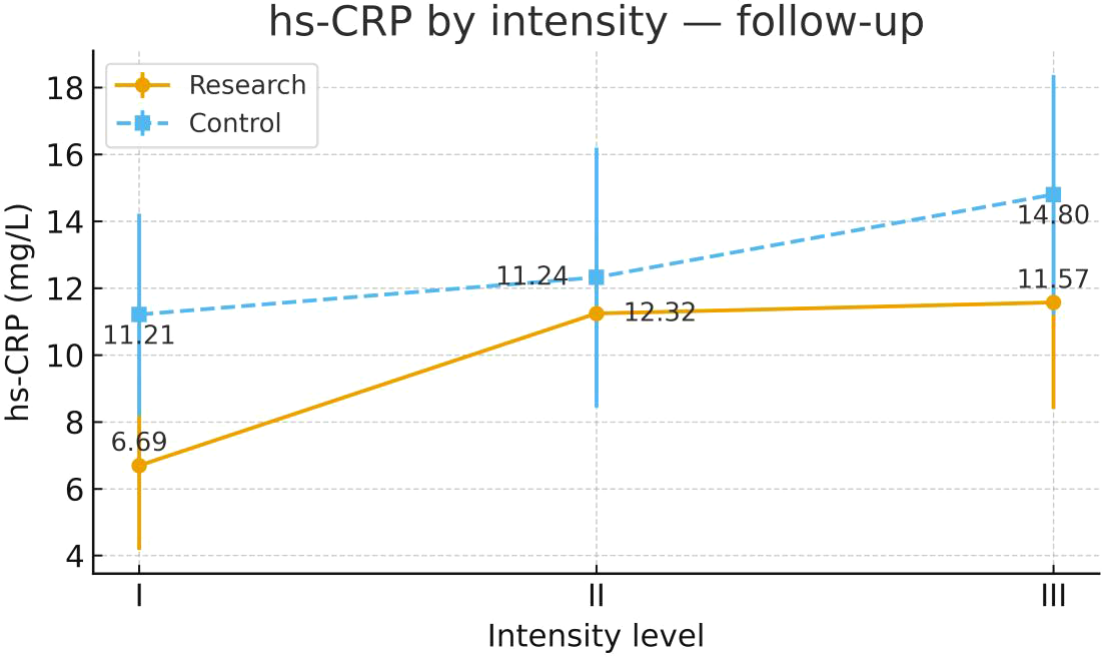
hs-CRP after intervention across severity strata.

**Figure 8:**
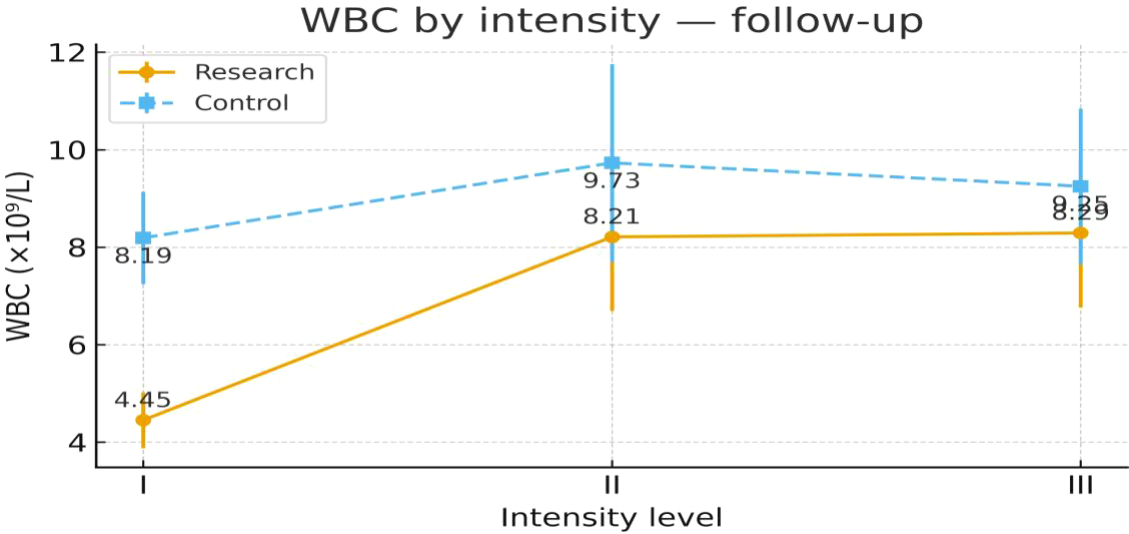
WBC after intervention across severity strata.

**Figure 9:**
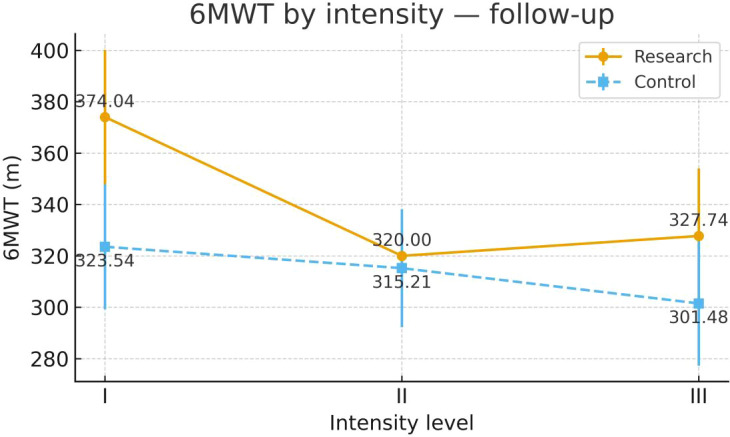
6MWT after intervention across severity strata.

**Figure 10:**
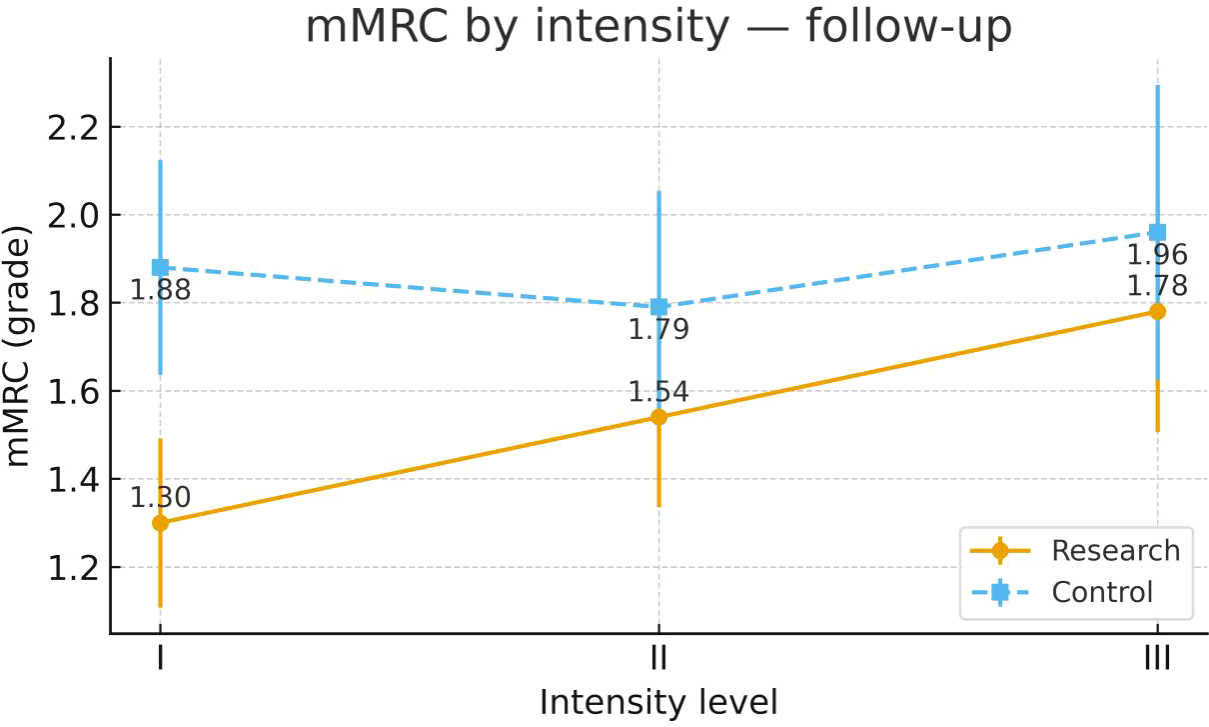
mMRC after intervention across severity strata.

**Figure 11:**
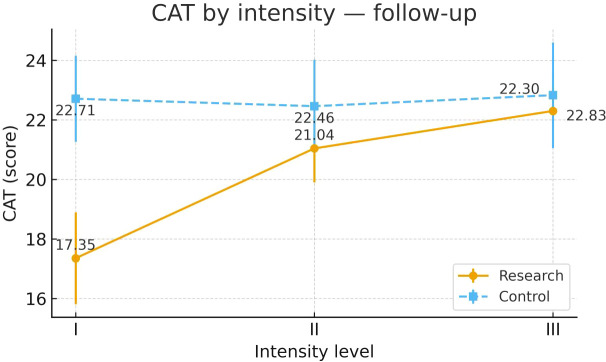
CAT after intervention across severity strata.

**Figure 12:**
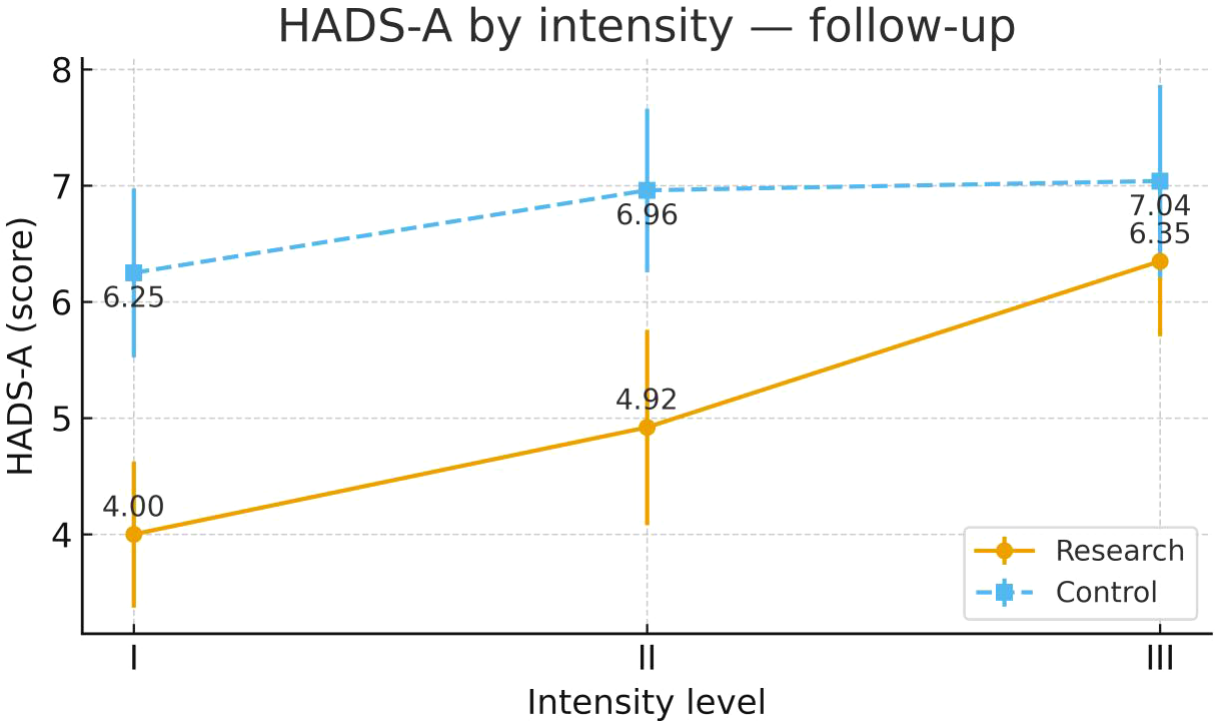
HADS-A after intervention across severity strata.

**Figure 13:**
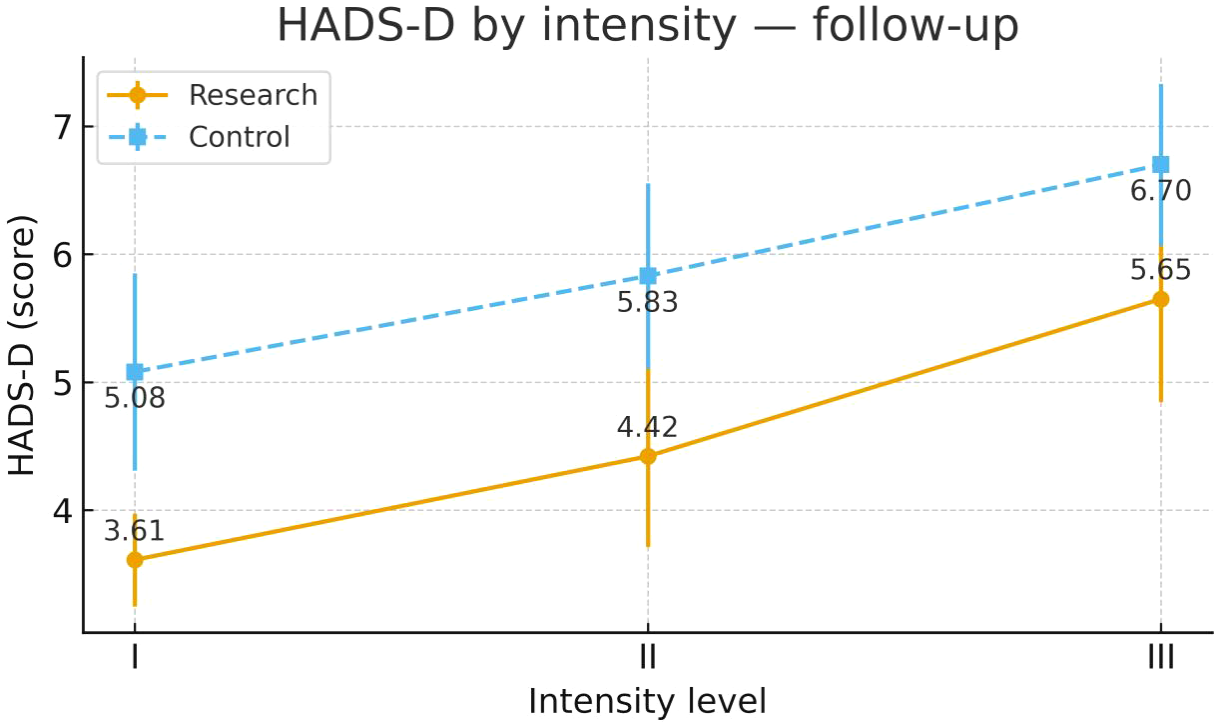
HADS-D after intervention across severity strata.

To examine heterogeneity of response under the severity-adapted prescription, post-intervention outcomes were compared within each group across the three prespecified baseline severity strata (Severity Grades I–III) (**Figures 4**–**13**). In the Study group, severity strata were linked to prescribed exercise intensity by inverse matching: Severity Grade I corresponded to Exercise Grade III, Severity Grade II to Exercise Grade II, and Severity Grade III to Exercise Grade I. Between-stratum differences in the Study group are therefore interpreted in the context of this severity-adapted matching. In the Control group, severity strata reflected baseline disease severity only, without intensity matching.

Across outcomes, several biomarkers and clinical endpoints showed clearer separation across strata in the Study group. The Control group did not show a consistent stratum pattern.

### 3.6 Analysis of efficacy differences across severity strata

To examine stratum-specific responses under inverse matching, subgroup analyses were performed across Severity Grades I–III. Results are presented in **Tables 8**–**17** and **Figures 4**–**13**.

**Table 8:** IL-6 before and after intervention across severity strata.

Group	Severity stratum	n	Mean ± SD (Pre-intervention)	F	P	Mean ± SD (Post-intervention)	F	P
Control Group	Severity Grade I	24	33.63 ± 8.06	0.322	0.726	28.15 ± 8.31	0.159	0.854
	Severity Grade II	24	35.56 ± 7.09			27.64 ± 8.71		
	Severity Grade III	23	35.22 ± 10.48			29.26 ± 11.12		
Study Group	Severity Grade I	23	34.17 ± 7.68	0.220	0.803	19.28 ± 4.36²³	7.315	0.001
	Severity Grade II	24	35.93 ± 11.04			27.42 ± 7.82¹		
	Severity Grade III	23	35.67 ± 9.90			27.86 ± 11.09¹		

**Table 9:** IL-8 before and after intervention across severity strata.

Group	Severity stratum	n	Mean ± SD (Pre-intervention)	F	P	Mean ± SD (Post-intervention)	F	P
Control Group	Severity Grade I	24	76.14 ± 31.27	0.687	0.507	79.69 ± 21.83	0.964	0.387
	Severity Grade II	24	80.90 ± 30.27			76.33 ± 24.55		
	Severity Grade III	23	87.45 ± 34.85			86.19 ± 22.25		
Study Group	Severity Grade I	23	82.60 ± 21.99	0.709	0.496	50.26 ± 19.77²³	5.371	0.007
	Severity Grade II	24	81.82 ± 33.49			71.17 ± 34.93¹		
	Severity Grade III	23	90.98 ± 29.92			75.34 ± 22.59¹		

**Table 10:** TNF-α before and after intervention across severity strata.

Group	Severity stratum	n	Mean ± SD (Pre-intervention)	F	P	Mean ± SD (Post-intervention)	F	P
Control Group	Severity Grade I	24	58.50 ± 14.51	0.192	0.826	53.90 ± 14.32	0.776	0.465
	Severity Grade II	24	60.26 ± 14.29			57.72 ± 16.82		
	Severity Grade III	23	57.77 ± 13.36			52.14 ± 12.68		
Study Group	Severity Grade I	23	58.01 ± 17.64	0.176	0.839	42.28 ± 8.05³	4.071	0.022
	Severity Grade II	24	59.87 ± 13.45			49.00 ± 12.50		
	Severity Grade III	23	57.36 ± 12.57			53.13 ± 15.76¹		

**Table 11:** hs-CRP before and after intervention across severity strata.

Group	Severity stratum	n	Mean ± SD (Pre-intervention)	F	P	Mean ± SD (Post-intervention)	F	P
Control Group	Severity Grade I	24	14.81 ± 10.19	0.384	0.683	11.21 ± 7.54	0.954	0.391
	Severity Grade II	24	16.52 ± 10.09			12.32 ± 9.71		
	Severity Grade III	23	17.27 ± 9.29			14.80 ± 8.77		
Study Group	Severity Grade I	23	13.65 ± 12.34	1.110	0.335	6.69 ± 6.16²³	3.648	0.031
	Severity Grade II	24	17.77 ± 11.64			11.24 ± 5.85¹		
	Severity Grade III	23	18.23 ± 10.46			11.57 ± 7.79¹		

**Table 12:** WBC before and after intervention across severity strata.

Group	Severity stratum	n	Mean ± SD (Pre-intervention)	F	P	Mean ± SD (Post-intervention)	F	P
Control Group	Severity Grade I	24	8.70 ± 2.85	0.776	0.464	8.19 ± 2.37	0.851	0.432
	Severity Grade II	24	10.13 ± 4.95			9.73 ± 5.08		
	Severity Grade III	23	9.51 ± 3.89			9.25 ± 3.91		
Study Group	Severity Grade I	23	8.80 ± 3.77	1.000	0.373	4.45 ± 1.40²³	9.600	P < 0.001
	Severity Grade II	24	9.54 ± 3.74			8.21 ± 3.79¹		
	Severity Grade III	23	10.45 ± 4.31			8.29 ± 3.73¹		

**Table 13:** 6MWT before and after intervention across severity strata.

Group	Severity stratum	n	Mean ± SD (Pre-intervention)	F	P	Mean ± SD (Post-intervention)	F	P
Control Group	Severity Grade I	24	271.29 ± 67.35	0.951	0.391	323.54 ± 60.80	0.830	0.440
	Severity Grade II	24	250.33 ± 50.61			315.21 ± 57.38		
	Severity Grade III	23	250.52 ± 61.81			301.48 ± 59.27		
Study Group	Severity Grade I	23	277.04 ± 47.87	1.851	0.165	374.04 ± 64.15²³	6.393	0.003
	Severity Grade II	24	256.58 ± 36.15			320.00 ± 33.69¹		
	Severity Grade III	23	255.52 ± 44.09			327.74 ± 64.46¹		

**Table 14:** mMRC before and after intervention across severity strata.

Group	Severity stratum	n	Mean ± SD (Pre-intervention)	F	P	Mean ± SD (Post-intervention)	F	P
Control Group	Severity Grade I	24	3.46 ± 0.83	0.587	0.559	1.88 ± 0.61	0.324	0.725
	Severity Grade II	24	3.67 ± 0.70			1.79 ± 0.66		
	Severity Grade III	23	3.70 ± 0.93			1.96 ± 0.82		
Study Group	Severity Grade I	23	3.35 ± 0.83	1.755	0.181	1.30 ± 0.47³	4.249	0.018
	Severity Grade II	24	3.54 ± 0.59			1.54 ± 0.51		
	Severity Grade III	23	3.74 ± 0.69			1.78 ± 0.67¹		

**Table 15:** CAT before and after intervention across severity strata.

Group	Severity stratum	n	Mean ± SD (Pre-intervention)	F	P	Mean ± SD (Post-intervention)	F	P
Control Group	Severity Grade I	24	26.17 ± 3.89	1.054	0.354	22.71 ± 3.63	0.053	0.948
	Severity Grade II	24	27.04 ± 3.42			22.46 ± 3.91		
	Severity Grade III	23	27.74 ± 3.85			22.83 ± 4.34		
Study Group	Severity Grade I	23	25.83 ± 2.23	1.399	0.254	17.35 ± 3.77²³	14.783	<0.001
	Severity Grade II	24	26.33 ± 3.00			21.04 ± 2.84¹		
	Severity Grade III	23	27.22 ± 3.23			22.30 ± 2.98¹		

**Table 16:** HADS-A before and after intervention across severity strata.

Group	Severity stratum	n	Mean ± SD (Pre-intervention)	F	P	Mean ± SD (Post-intervention)	F	P
Control Group	Severity Grade I	24	10.13 ± 2.23	0.495	0.612	6.25 ± 1.82	1.287	0.283
	Severity Grade II	24	9.83 ± 1.49			6.96 ± 1.76		
	Severity Grade III	23	10.39 ± 1.97			7.04 ± 2.03		
Study Group	Severity Grade I	23	9.13 ± 1.89	1.438	0.245	4.00 ± 1.54³	10.326	<0.001
	Severity Grade II	24	9.54 ± 2.67			4.92 ± 2.10³		
	Severity Grade III	23	10.26 ± 2.22			6.35 ± 1.58¹²		

**Table 17:** HADS-D before and after intervention across severity strata.

Group	Severity stratum	n	Mean ± SD (Pre-intervention)	F	P	Mean ± SD (Post-intervention)	F	P
Control Group	Severity Grade I	24	9.33 ± 1.88	0.442	0.645	5.08 ± 1.93³	4.854	0.011
	Severity Grade II	24	9.67 ± 1.76			5.83 ± 1.81		
	Severity Grade III	23	9.83 ± 1.88			6.70 ± 1.55¹		
Study Group	Severity Grade I	23	9.39 ± 2.08	0.016	0.984	3.61 ± 0.89³	9.356	<0.001
	Severity Grade II	24	9.50 ± 2.32			4.42 ± 1.77³		
	Severity Grade III	23	9.48 ± 2.06			5.65 ± 1.97¹²		

General notes for **Tables 8**-**17**: Data are mean ± SD. Within each group, differences across severity strata were tested using one-way ANOVA; F denotes the ANOVA omnibus F statistic and P denotes its corresponding significance level. Superscripts indicate significant *post-hoc* pairwise differences (LSD, P < 0.05): ¹ vs Severity Grade I, ² vs Severity Grade II, ³ vs Severity Grade III.

For consistency, all outcomes are presented as mean ± SD at baseline (pre-intervention) and post-intervention in **Table 8**–**17**, and the text summarizes direction and between-stratum patterns without repeating numeric values.

#### 3.6.1 IL-6

Post-intervention IL-6 differed across severity strata in the Study group. Severity Grade I showed the most favorable post-intervention profile. Severity Grades II and III were broadly similar. No stratum-related differences were observed in the Control group. Detailed results are shown in **Table 8** and **Figure 4**.

#### 3.6.2 IL-8

Post-intervention IL-8 differed across severity strata in the Study group. Severity Grade I showed a more favorable post-intervention profile than Severity Grades II and III. Severity Grades II and III were similar. No stratum-related differences were observed in the Control group. Detailed results are shown in **Table 9** and **Figure 5**.

#### 3.6.3 TNF-α

Post-intervention TNF-α differed across severity strata in the Study group. Severity Grade I showed a more favorable profile than Severity Grade III. The difference between Severity Grades I and II did not reach statistical significance. No stratum-related differences were observed in the Control group. Detailed results are shown in **Table 10** and **Figure 6**.

#### 3.6.4 hs-CRP

Post-intervention hs-CRP differed across severity strata in the Study group. Severity Grade I showed greater improvement than Severity Grades II and III. Severity Grades II and III were similar. No stratum-related differences were observed in the Control group. Detailed results are shown in **Table 11** and **Figure 7**.

#### 3.6.5 WBC

Post-intervention WBC showed a clear stratum pattern in the Study group. Severity Grade I showed a more pronounced improvement than Severity Grades II and III. No stratum-related differences were observed in the Control group. Detailed results are shown in **Table 12** and **Figure 8**.

#### 3.6.6 6MWT

Post-intervention 6MWT differed across severity strata in the Study group. Severity Grade I showed greater improvement than Severity Grades II and III. Severity Grades II and III did not show clear separation. No stratum-related differences were observed in the Control group. Detailed results are shown in **Table 13** and **Figure 9**.

#### 3.6.7 mMRC

Post-intervention mMRC differed across severity strata in the Study group. Severity Grade I showed a more favorable dyspnea profile than Severity Grade III. Comparisons involving Severity Grade II did not reach statistical significance. No stratum-related differences were observed in the Control group. Detailed results are shown in **Table 14** and **Figure 10**.

#### 3.6.8 CAT

Post-intervention CAT differed across severity strata in the Study group. Severity Grade I showed greater improvement than Severity Grades II and III. Severity Grades II and III were similar. No stratum-related differences were observed in the Control group. Detailed results are shown in **Table 15** and **Figure 11**.

#### 3.6.9 HADS-A

Post-intervention HADS-A differed across severity strata in the Study group. Severity Grades I and II showed comparable improvement. Both were more favorable than Severity Grade III. No stratum-related differences were observed in the Control group. Detailed results are shown in **Table 16** and **Figure 12**.

#### 3.6.10 HADS-D

Post-intervention HADS-D differed across severity strata in the Study group. Severity Grades I and II showed more favorable improvement than Severity Grade III. Severity Grades I and II were broadly similar. In the Control group, a mild stratum-related difference was observed. Detailed results are shown in **Table 17** and **Figure 13**.

## 4 Discussion

improvements in exercise tolerance, dyspnea, health status, and psychological scores than conventional rehabilitation. In this assessor-blinded randomized trial, a severity-adapted graded exercise program delivered during the hospitalization period was associated with larger short-term improvements in the inflammatory marker profile and greater. Among the inflammatory outcomes, the most statistically robust between-group differences were observed for IL-8, TNF-α, and WBC, whereas IL-6 and hs-CRP showed directionally consistent but more modest evidence after accounting for multiplicity (Spruit et al., 2013; Thompson et al., 2013; Puhan et al., 2016; Rochester et al., 2023; Newman et al., 2024; Patel, 2024).

Previous studies indicate that early rehabilitation alone does not guarantee improvement across all endpoints. In-hospital programs may accelerate recovery of physical function, whereas longer-term outcomes such as readmission remain inconsistent, suggesting that the training stimulus, program structure, and implementation quality are important. Meta-analyses in AECOPD also report heterogeneity for dyspnea, quality-of-life measures, and mortality, emphasizing the need for structured prescriptions and safety monitoring when rehabilitation is delivered during the acute inpatient phase (Greening et al., 2014; Puhan et al., 2016; Mohammed et al., 2018; Li et al., 2021).

Regarding inflammatory markers, prior syntheses suggest that pulmonary rehabilitation can reduce TNF-α and CRP compared with usual care, while findings for IL-6 are less consistent (Thompson et al., 2013; Li et al., 2021). Our results add evidence from an inpatient AECOPD setting using a severity-adapted prescription. Because systemic inflammation and symptoms often decline rapidly during hospitalization under standard treatments, the observed reductions should be interpreted in the context of concurrent medical recovery. The additional benefit seen in the graded program likely reflects earlier mobilization and a more structured training stimulus during this recovery window, rather than long-term structural remodeling. Within two weeks, the improvements are more consistent with reduced deconditioning, better symptom control, and increased tolerance to exertion.

A key feature of this trial is the prespecified inverse matching between severity grade and exercise intensity grade. While guidelines emphasize individualized prescription, direct evidence defining optimal intensity ranges across severity strata remains limited (Spruit et al., 2013; Patel, 2024). Under predefined monitoring and suspension criteria, clinically stable patients with milder exacerbations tolerated a more active stimulus, whereas patients with greater severity achieved gains with lower-to-moderate intensity within safe limits. To reduce the possibility that subgroup patterns merely reflect baseline severity, we reported baseline inflammatory and functional measures within each severity stratum, which showed no meaningful between-group imbalances. In addition, post-intervention separation across strata was not consistently observed in the Control group, supporting the interpretation that the graded prescription contributed to the observed stratum patterns. These findings are compatible with an intensity–response rationale under individualized dosing, but they should be viewed as supportive rather than definitive, because intensity was assigned by the matching rule rather than randomized by intensity level. From a physiological standpoint, short-term gains during hospitalization likely reflect improved symptom control, reduced ventilatory limitation during activity, and attenuation of bed-rest–related deconditioning (Mohammed et al., 2018).

The improvement in HADS-A and HADS-D over the hospitalization period should also be interpreted within the inpatient context. Anxiety and depressive symptoms in AECOPD can improve as dyspnea is relieved and activity becomes less threatening. Supervised sessions, monitoring, and structured guidance may further reduce fear associated with exertion. The observed psychological benefits therefore likely reflect both symptom improvement during recovery and supportive inpatient care alongside the exercise intervention.

Several directions should be considered for future research. Longer follow-up is needed to determine whether the short-term inpatient benefits persist after discharge and translate into outcomes such as readmission and sustained quality of life. Multicenter trials with standardized reporting of intensity, volume, frequency, and monitoring procedures would improve reproducibility, and objective workload metrics would help clarify dose–response relationships (Puhan et al., 2016; Li et al., 2021). More refined stratification models incorporating inflammatory phenotypes, muscle strength or mass, comorbidity burden, and psychological load may improve progression algorithms (Pedersen and Febbraio, 2008; Mohammed et al., 2018; Li et al., 2021). Programs that bridge inpatient, outpatient, and home-based rehabilitation may further clarify long-term effectiveness (Greening et al., 2014; Mohammed et al., 2018; Li et al., 2021).

In summary, within the constraints of short-term inpatient follow-up, a severity-adapted graded exercise rehabilitation strategy was associated with a more favorable inflammatory profile and better functional and symptom outcomes than conventional rehabilitation (Thompson et al., 2013; Greening et al., 2014; Puhan et al., 2016; Li et al., 2021; Patel, 2024).

### 4.1 Study Limitations

This study has several limitations. First, all participants received standard pharmacologic treatment for AECOPD during hospitalization, so biomarker changes occurred during routine medical recovery and the independent contribution of exercise cannot be fully isolated. In addition, detailed medication exposure, such as corticosteroid dose, antibiotic regimen, and bronchodilator intensity, was not modeled in the primary analysis. Although randomization and management within the same inpatient setting would be expected to reduce major systematic imbalance, residual confounding by unmeasured pharmacotherapy cannot be excluded. Second, outcome assessors were blinded, but rehabilitation staff could not be blinded, which may have influenced effort-dependent outcomes; this risk was reduced by standardized testing procedures conducted by blinded assessors. Third, although actual delivered dose was reported using session-based metrics, including planned sessions, completed sessions, completion rate, down-titration, and early termination, objective external workload measures were not available. Training intensity was prescribed and adjusted using heart rate, symptoms, and oxygen saturation, which limits precise quantification of training dose and reduces cross-study comparability. Fourth, although the severity-stratified analyses were prespecified, the sample size within each stratum limits certainty; these subgroup patterns should be interpreted as exploratory and confirmed in larger studies. Finally, outcomes were assessed over a short inpatient period, and persistence after discharge remains uncertain. Future multicenter studies with longer follow-up and objective workload monitoring are needed to refine the prescription algorithm and strengthen external validity.

## 5 Conclusion

In this prospective, randomized controlled, assessor-blinded trial, a severity-adapted graded exercise rehabilitation program initiated during hospitalization for AECOPD was associated with a more favorable inflammatory profile and greater improvements in functional capacity, dyspnea, health status, and psychological outcomes than conventional rehabilitation. Subgroup findings further suggested that clinically stable patients with milder disease and better physiological reserve tended to derive greater benefit from a relatively higher, well-tolerated training stimulus. These findings support the integration of a severity- and function-informed intensity-matching approach into inpatient rehabilitation pathways, with session-to-session adjustment based on clinical status and tolerance.

## Data Availability

The data analyzed in this study is subject to the following licenses/restrictions: no. Requests to access these datasets should be directed to zenghui.1221@163.com.

## Supplementary material

Supplementary Table 1 Severity-adapted graded exercise rehabilitation protocol, monitoring, and stopping criteria for hospitalized AECOPD patients.

Supplementary Appendix 1 Emergency management procedures during rehabilitation sessions.
