# Supplementary material for: Severity-adapted graded exercise rehabilitation reduces systemic inflammation and improves functional capacity in hospitalized AECOPD: an assessor-blinded randomized controlled trial
Source: Front Physiol. 2026 Apr 16;17:1767608. doi: 10.3389/fphys.2026.1767608 (PMC13128363; doi:10.3389/fphys.2026.1767608)
Supplement: Supplementary Table 1 — Severity-adapted graded exercise rehabilitation protocol, monitoring, and stopping criteria for hospitalized AECOPD patients. [file Table1.docx]

# Supplementary Table S1

**Severity-adapted graded exercise rehabilitation protocol, monitoring, and stopping criteria for hospitalized AECOPD patients.**

## Section A. Pre-exercise assessment and timing

| Item | Description |
| --- | --- |
| Assessment timing | Before the first exercise session; before every subsequent session; and whenever clinical condition changes or the exercise prescription is modified |
| Clinical assessment | Medical history related to current admission; symptoms and signs including dyspnea, cough, sputum, wheeze, chest tightness, appetite, and body weight |
| Physiological assessment | Vital signs, level of consciousness, oxygenation status, and relevant laboratory and imaging information available during hospitalization |
| Functional assessment | Borg Dyspnea Scale, 6-minute walk test (6MWT), activities of daily living, and COPD Assessment Test (CAT) score |
| Assessment methods | Direct observation, patient and family inquiry, physical examination, and review of electronic medical and nursing records |

## Section B. Session-level contraindications (before exercise)

| Category | Contraindications |
| --- | --- |
| Hemodynamic instability | Uncontrolled hypertension (>180/110 mmHg) or hypotension (<90/60 mmHg) |
| Cardiovascular conditions | Unstable angina, severe arrhythmias, recent myocardial infarction, congestive heart failure, or uncontrolled pulmonary hypertension (mean pulmonary artery pressure >55 mmHg) |
| Pulmonary and systemic conditions | Acute bronchial asthma exacerbation, pulmonary embolism, pneumothorax, hemoptysis, metastatic malignancy |
| Neuromuscular and musculoskeletal limitations | Conditions affecting safe exercise performance, including neuromuscular disorders, bone or joint disease, limb disability, or deep vein thrombosis |

## Section C. Severity–intensity matching and graded exercise prescriptions

| **Grade I exercise (low intensity; prescribed for disease severity Grade III)** | |
| --- | --- |
| Component | Description |
| Eligibility profile | RR >24 breaths/min; accessory muscle use; oxygen concentration >40% required to correct hypoxemia; PaCO₂ >60 mmHg or presence of acidosis (pH ≤7.25); acute mental status change with ability to cooperate |
| Respiratory training | Relaxed, regular breathing to reduce ventilatory load and improve patient–ventilator synchrony when applicable |
| Exercise training | Passive-to-active progression as tolerated; multisensory stimulation; basic upper- and lower-limb mobility exercises |
| Dose | 2–3 sets per exercise; 8–12 repetitions per set, adjusted according to tolerance |
| **Grade II exercise (moderate intensity; prescribed for disease severity Grade II)** | |
| Component | Description |
| Eligibility profile | RR >24 breaths/min; accessory muscle use; oxygen concentration >35% required; PaCO₂ increased from baseline or 50–60 mmHg; no alteration in mental status |
| Respiratory muscle training | Pursed-lip diaphragmatic breathing; 5–10 min per session |
| Exercise training | Assisted active limb training, bed mobility, strengthening exercises, and transfer training as clinical stability improves |
| Dose | 2–3 sets per exercise; 8–12 repetitions per set |
| **Grade III exercise (higher intensity; prescribed for disease severity Grade I)** | |
| Component | Description |
| Eligibility profile | RR ≤24 breaths/min; HR <95 beats/min; no accessory muscle use; hypoxemia corrected with oxygen concentration 24–35%; no increase in PaCO₂ |
| Respiratory muscle resistance training | Resisted diaphragmatic breathing with progressive external load (0.5–2 kg) and/or expiratory resistance tasks; 3–5 min per session |
| Resistance training | Upper- and lower-limb resistance training with progressive external load (0.5–2 kg) |
| Aerobic training | Walking and stair training with progression guided by tolerance |
| Dose | 2–3 sets per exercise; 8–12 repetitions per set |

## Section D. Exercise intensity prescription and adjustment rules

| Parameter | Prescription |
| --- | --- |
| Target heart rate (THR) | THR = [(220 − age) − resting heart rate] × (60%–80%) + resting heart rate |
| Borg dyspnea target | 4–6 |
| Load adjustment | Increase load or volume if Borg <4; reduce load or volume if Borg >6 |
| Progression principle | Stepwise progression from lower to higher intensity based on tolerance |

## Section E. Session structure, frequency, and initiation criteria

| Item | Description |
| --- | --- |
| Initiation criteria | Clinically stable; SpO₂ ≥90%; blood pressure ≥90/60 mmHg |
| Session structure | Warm-up (5–10 min), intermittent exercise with rest as needed, and cool-down (5–10 min) |
| Exercise pattern | Intermittent; rest 2–3 min when fatigue occurs |
| Frequency | Twice daily |
| Duration | Duration: 30–45 min per session, delivered intermittently and including rest intervals (warm-up and cool-down prescribed separately when feasible) |
| Intervention period | Up to 2 weeks or until discharge |

## Section F. Monitoring and exercise suspension criteria

| Category | Suspension criteria |
| --- | --- |
| Cardiac | Arrhythmia, acute heart failure, acute myocardial infarction; heart rate exceeding upper THR limit for >3 min |
| Respiratory | RR <5 or >30 breaths/min; persistent dyspnea >3 min; SpO₂ <85% for >3 min; patient–ventilator asynchrony during non-invasive ventilation |
| Blood pressure | BP >180/110 mmHg or <90/60 mmHg; MAP <65 mmHg; ≥20% change from baseline BP lasting >3 min; initiation or dose increase of vasoactive drugs |
| Other | Chest tightness or pain, dizziness, profuse sweating, pallor, inability or refusal to continue exercise |

## Section G. Daily evaluation and progression

| Item | Description |
| --- | --- |
| Evaluation timing | Daily after intervention |
| Evaluation domains | Dyspnea, pulmonary function, exercise capacity, and quality of life |
| Evaluation tools | mMRC Dyspnea Scale, pulmonary function testing, 6-minute walk test (6MWT), and COPD Assessment Test (CAT) |
| Adjustment | Exercise prescription modified when insufficient improvement or intolerance is observed |
