# Supplementary material for: Severity-adapted graded exercise rehabilitation reduces systemic inflammation and improves functional capacity in hospitalized AECOPD: an assessor-blinded randomized controlled trial
Source: Front Physiol. 2026 Apr 16;17:1767608. doi: 10.3389/fphys.2026.1767608 (PMC13128363; doi:10.3389/fphys.2026.1767608)
Supplement: Supplementary Appendix 1 — Emergency management procedures during rehabilitation sessions. [file Table2.docx]

# Supplementary Appendix S1

## Emergency Management Procedures During Rehabilitation Sessions

**Purpose and scope**

This appendix describes the standardized emergency management procedures applied during in-hospital rehabilitation sessions for patients with acute exacerbation of chronic obstructive pulmonary disease (AECOPD). The procedures were designed to ensure patient safety during graded exercise training by enabling rapid recognition of intolerance, prompt clinical assessment, timely escalation of care, and consistent documentation. These procedures complemented routine physiological monitoring and the predefined criteria for suspending exercise described in Supplementary Table S1.

**General safety principles**

**Across all rehabilitation sessions, the following principles were implemented:**

Immediate interruption of exercise: Any sign of acute intolerance or suspected adverse event prompted immediate cessation of the ongoing activity. Patients were assisted into a safe position (sitting or supine as clinically appropriate) and reassured to minimize anxiety-related ventilatory load.

Rapid clinical assessment and stabilization: Rehabilitation staff performed an immediate assessment focused on airway patency, breathing pattern, circulation, and neurological status. Vital signs were obtained promptly, and oxygen supplementation was adjusted according to clinical need and physician guidance.

Timely escalation and physician notification: The responsible physician was notified without delay for events suggestive of cardiopulmonary instability, neurological change, or injury requiring medical evaluation. The institutional emergency response pathway was activated when indicated.

Event documentation and follow-up: The event type, onset context, symptoms, vital signs, management steps, and outcome were recorded in a standardized manner. Decisions regarding resumption or modification of rehabilitation were made after clinical reassessment and, when necessary, physician clearance.

Return-to-exercise strategy: Following stabilization, rehabilitation was resumed only when the patient met predefined safety criteria and demonstrated tolerance. Training intensity and volume were reduced, and progression was delayed when events suggested limited physiological reserve.

**Event-specific procedures**

**1) Acute severe dyspnea or respiratory decompensation**

Typical presentation: Sudden or rapidly worsening dyspnea disproportionate to the exercise load, persistent tachypnea, inability to speak in full sentences, signs of increased work of breathing, marked anxiety, or deterioration in oxygenation during a session.

**Immediate management:**

Stop exercise immediately and assist the patient into a position that reduces ventilatory load.

Assess airway patency and breathing effort; obtain oxygen saturation and respiratory rate promptly.

Provide oxygen supplementation and supportive breathing guidance. For patients receiving non-invasive ventilation, staff prioritized patient–ventilator synchrony and ensured interface positioning and comfort.

If symptoms persisted despite initial measures or clinical deterioration was suspected, the responsible physician was notified for urgent evaluation and escalation of therapy.

**Follow-up:**

Rehabilitation was withheld until clinical stability was re-established. When training resumed, the subsequent session used a lower initial intensity and shorter work intervals with longer rest periods, with close monitoring.

**2) Suspected acute coronary syndrome or clinically significant chest symptoms**

Typical presentation: New-onset chest pain or tightness, pain radiating to the jaw/arm, diaphoresis, pallor, syncope or near-syncope, marked tachycardia or bradycardia, or other symptoms suggestive of myocardial ischemia during or immediately after exercise.

**Immediate management:**

Stop exercise and place the patient at rest in a safe position.

Obtain a rapid clinical assessment, including vital signs and symptom characterization, and initiate continuous monitoring when available.

Notify the responsible physician immediately for urgent medical evaluation and management, and activate the institutional emergency response pathway if indicated.

Cardiopulmonary resuscitation and defibrillation readiness were maintained according to hospital protocols for any patient with hemodynamic instability or loss of consciousness.

**Follow-up:**

Rehabilitation remained suspended until medical evaluation was completed and physician clearance was obtained. Any return to exercise followed a conservative re-initiation plan with reduced intensity and enhanced monitoring.

**3) Soft tissue injury related to training**

Typical presentation: Localized pain, swelling, reduced range of motion, or functional limitation occurring during a movement task or transfer activity, without signs of systemic compromise.

**Immediate management:**

Stop the provoking activity and protect the affected area.

Perform a brief assessment of pain severity, range of motion, and functional impact; inspect for signs suggesting fracture or significant structural injury.

Notify clinical staff if symptoms were severe, progressive, or raised concern for more than minor injury.

Provide symptom-directed supportive management as per institutional practice and physician guidance.

**Follow-up**:

Subsequent training avoided provocative movements and emphasized pain-free range of motion, gradual reintroduction of resistance, and functional tasks consistent with safe tolerance.

**4) Muscle cramps during exercise**

Typical presentation: Sudden involuntary muscle contraction with localized pain, commonly in lower limbs during active training or transfer tasks.

**Immediate management:**

Stop exercise and assist the patient into a stable position.

Apply gentle, slow stretching of the affected muscle group and allow adequate recovery time.

Reassess for contributing factors such as fatigue, insufficient warm-up, or excessive load for the current session.

**Follow-up:**

When training resumed, the session proceeded with reduced intensity and emphasis on proper warm-up, gradual loading, and sufficient rest intervals. Recurrent cramps prompted review of the prescription and hydration/nutrition status under clinical guidance.

**5) Falls or fall-related injury**

Typical presentation: Loss of balance during transfer, ambulation, or bedside tasks, with or without visible injury; possible transient loss of consciousness or confusion.

**Immediate management:**

Stop all activity immediately and ensure scene safety.

Assess level of consciousness, symptoms (e.g., headache, dizziness), and any visible injury.

Obtain vital signs and perform a focused neurological and musculoskeletal screening.

Notify the responsible physician promptly for further evaluation, particularly if head impact, persistent pain, neurological symptoms, or hemodynamic instability was suspected.

Assist with safe transfer and continued monitoring as clinically appropriate.

**Follow-up:**

Rehabilitation was resumed only after clinical reassessment. Subsequent sessions prioritized fall-risk mitigation through enhanced assistance, simplified tasks, and conservative progression.

**Documentation and quality assurance**

All adverse events or suspected intolerance episodes were documented using standardized fields, including event classification, temporal relationship to exercise, vital signs, immediate management measures, and clinical outcomes. Rehabilitation prescriptions were reviewed after each event, and progression was modified to prioritize safety while maintaining participation in tolerable training components. Event summaries were incorporated into routine team quality review to ensure consistent implementation of safety procedures.
